# A positive approach to adolescent sexual health promotion: a qualitative evaluation of key stakeholder perceptions of the Australian Positive Adolescent Sexual Health (PASH) Conference

**DOI:** 10.1186/s12889-019-6993-9

**Published:** 2019-06-03

**Authors:** Brianna C. S. Crocker, Sabrina W. Pit, Vibeke Hansen, Franklin John-Leader, Mary Louise Wright

**Affiliations:** 1 School of Medicine, University Of Wollongong, Wollongong, Australia; 2 University Centre for Rural Health, School of Medicine, Western Sydney University, 62 Uralba Street, POBox 3074, Lismore, NSW 2480 Australia; 3 Sydney School of Public Health, University Of Sydney, Sydney, Australia; 4 Harm Reduction and Health Promotion Programs, HIV and Related Programs (HARP), North Coast Public Health, Mid-North Coast Local Health District, Lismore, Australia

**Keywords:** Adolescents, Sexual health, Wellbeing, Positive youth development, Health promotion

## Abstract

**Background:**

Positive youth development (PYD) models are effective in improving adolescent sexual health. Adolescent programs including peer educators, parents and the wider community also demonstrate effectiveness in improving sexual health outcomes. An innovative Positive Adolescent Sexual Health (PASH) Conference model has been introduced in Northern NSW, Australia. It is run by the North Coast PASH Consortium, which is based on a health promotion framework. It takes a positive and holistic approach to sexual health education, and incorporates peer educators, parents, community workers and teachers. This study provides an introductory evaluation of the PASH Conference and identifies areas for increased effectiveness. It is intended as an early piece of research to inform future evaluations and to provide introductory information for public health educators.

**Methods:**

Data collection included semi-structured interviews with 13 key stakeholders of the PASH Conference. Interviews were recorded, transcribed verbatim and analysed using deductive thematic analysis.

**Results:**

Subjects included 2 teachers, 2 parents, 2 youth conference workers, 2 organisers, 2 presenters and 3 Peer Educators engaging Peers (PEEPs). Stakeholders perceived that young people were engaged to strengthen their sexual health and wellbeing due to many factors. These followed 3 themes: a safe and open learning environment, empowerment of young people and involvement of the support system and broader community. Multiple recommendations were identified across 2 themes: changes to conference format and planning, and enhancing stakeholder engagement.

**Discussion:**

The PASH Conference is a promising new youth development design promoting positive adolescent sexual health, which may provide a feasible model for public health educators to trial. Elements of the conference identified as engaging to youth align well with those in PYD research literature. This study provides an early piece of research to inform the design of future research on the PASH Conference including evaluation of behavioural outcomes. It provides introductory information to inform PASH Conference development to further increase its effectiveness.

**Electronic supplementary material:**

The online version of this article (10.1186/s12889-019-6993-9) contains supplementary material, which is available to authorized users.

## Background

Research suggests that adolescents require support in gaining skills and knowledge for healthy sexual development and behaviour [1, 2]. A nationally representative Australian survey demonstrated that 13% of sexually active high-school students report using no contraception the last time they engaged in intercourse, 15% use the ‘withdrawal method’ and just over half use a condom [2]. Adolescence is also a period of increased risk of peer pressure and sexual coercion [3]. An experience of unwanted sex was reported by 25% of students and over half had received sexually explicit text messages [2]. Adolescents require knowledge and skills to negotiate healthy, non-exploitative sexual relationships [4].

Sex education is a primary strategy for achieving improved adolescent sexual health outcomes [5]. School-based programs have traditionally been the main source [4]. However 50% of young Australians express significant dissatisfaction with school sex education citing irrelevance to real-life experience and inadequate discussion of important issues, e.g. positive sexual relationships and consent [2]. Furthermore, students prefer community health or peer educators to teach sex education rather than school teachers [2, 6].

Programs taking a holistic, positive approach to youth sexuality and development have demonstrated effectiveness. The Brazilian *PEAS Belgo* program emphasises relationships, a healthy sex life and gender equity. Run by health professionals, it includes peer education, family and local involvement, and has demonstrated improvements in sexual health outcomes [7]. Skills training programs also demonstrate effectiveness, e.g. *Smart Girls*: North Carolina, which provides positive life skills strategies, had increased personal and self-sexuality expectations compared to control groups [8]. Sexual health models incorporating parents frequently demonstrate effectiveness [9–12].

Programs incorporating peer education are popular. It is generally believed that a peer-led approach is appropriate since adolescents have a greater propensity to be influenced by and learn from their peers [13]. However, whilst peer education appears to be effective in increasing knowledge and changing attitudes, there is a lack of consensus on their impact on outcomes [14–18].

Positive youth development (PYD) programs promoting adolescent sexual health behaviours differ from sex education programs in that they focus more on strengthening wellbeing, skills and relationships, with sexual health incorporated within these [19–21]. Many PYD programs have shown to strengthen adolescent sexual health [20]. PYD programs deemed effective have key characteristics. These include delivering activities in a positive, supportive environment, strengthening the school and family context, empowering youth, building skills and engaging youth in real activities and roles [19, 20].

On the North Coast of NSW Australia, a voluntary consortium named the Positive Adolescent Sexual Health (PASH) Consortium has been developed. The PASH Consortium convenes annual conferences to improve adolescent sexual health. The Consortium and the strategies they implement are based on the health promotion model set out in the World Health Organization’s (WHO) Ottawa Charter for Health Promotion [22]. Health promotion, according to the Ottawa Charter, includes multiple elements such as providing a supportive environment, strengthening community actions, developing personal skills and reorienting health services to focus on the needs of the individual as a whole [22]. The PASH Conference is based on the health promotion model as well as the PYD framework, and aims to take a positive approach to improve young people’s overall wellbeing including sexual health. It also incorporates peer education, parental, teacher and community involvement.

There is a paucity of Australian research evaluating adolescent sexual health programs that 1) include all relevant actors e.g. peer educators, parents, community workers and teachers 2) take a positive approach based on a positive youth development and health promotion framework and 3) follow a conference format. Hence, informative research is needed to address these gaps. This is important to identify new ways of promoting positive adolescent sexual health. This study will provide a broad overview of the conference, with the intention that it will inform the design of future in-depth qualitative analyses, and quantitative studies assessing behavioural outcomes. Additionally, this study will also provide introductory information for public health practitioners, enabling the trial of an innovative Positive Youth Development model promoting sexual health. Therefore, our research aims are to:

1. provide an introductory exploration among key stakeholders of their perceptions on how the PASH Conference engages young people to strengthen their sexual health and wellbeing.
2. identify potential recommendations from key stakeholders to improve effectiveness and efficiency of future PASH Conferences.

## Methods

### Intervention

**Fig. 1:**
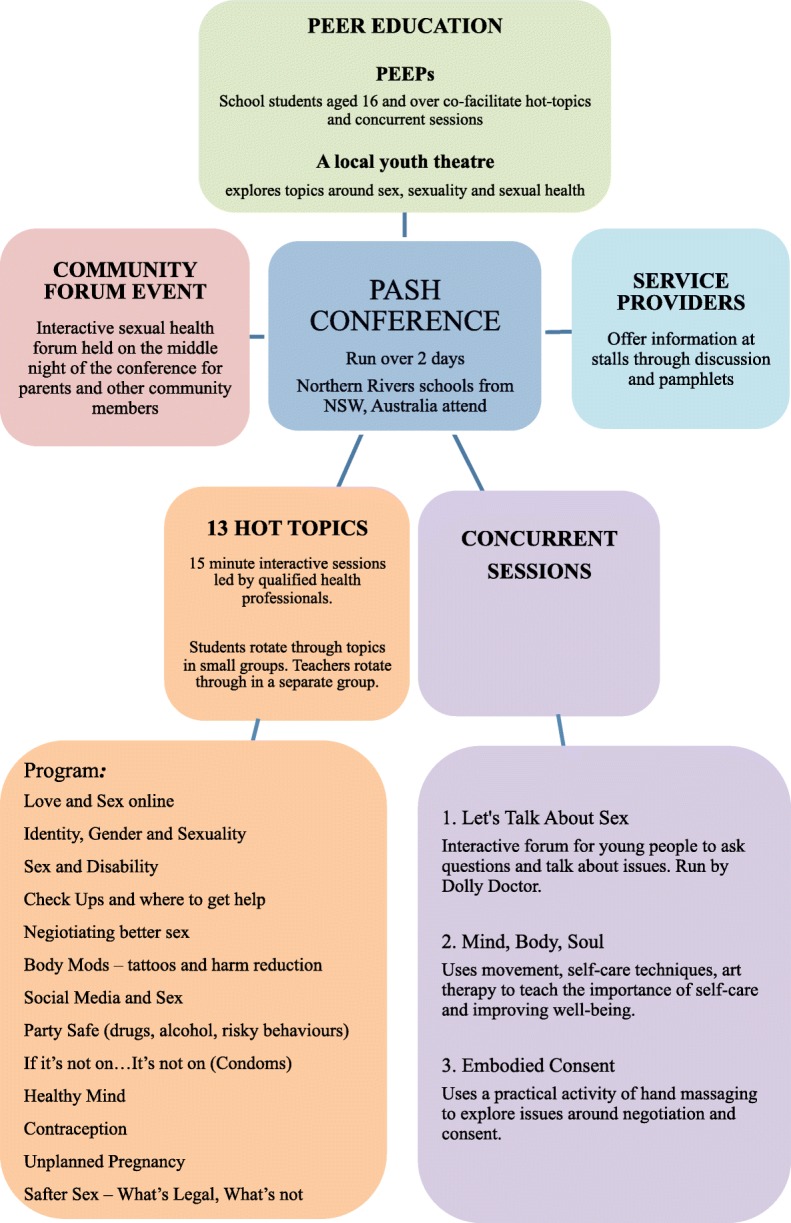
Elements of the PASH Conference

The PASH Conference has taken place annually on the North Coast of NSW, Australia since 2014. Figure 1 provides an overview of the conference. Conducted over 2 days, it aims to “enable young people aged 15 and over to build their confidence, skills, resilience and knowledge of sex, sexuality and sexual health services and related issues” [23]. It is organised by the North Coast PASH Consortium, a voluntary network of 30 North Coast youth, education and health organisations [23].

The conference incorporates many tools with the aim of developing healthy behaviours and skills around wellbeing, sexual health, sexuality, body image and more. Young people are involved in planning and engagement as are parents, professionals, teachers and community members.

All high schools across Northern NSW, from Grafton to Tweed Heads, were invited to register a group of students to attend the PASH Conference. At least one teacher from each participating school attended, and this included Personal Development, Health and Physical Education teachers, year advisors, school counsellors and chaplains. Peer educators (PEEPs) were also invited (16–24 year olds) to attend a two-day training program prior to the conference from all regional high schools and youth services across the Northern NSW region. The PASH conference as well as the PEEPs training was widely promoted via school newsletters, various youth inter-agencies and youth services across Northern NSW. In total 18 schools participated, with 720 young people, 120 teachers, youth workers and parents attending over the two days.

### Study design

Qualitative semi-structured interviews took place in Northern NSW, Australia, from December 2016 to February 2017. Whilst the majority of interviews (10) were conducted in December 2016, three were conducted in January and February 2017 due to limited availability of these interviewees. There did not appear to be any difference in the quality of recollections or discussion for those interviewed at a later date.

#### Data collection

A semi-structured interview guide was developed to facilitate discussion around the overall aims. The interview guide (Additional file 1) was developed by the primary researcher in association with an advisory group consisting of two academics, two child psychologists and a social marketing expert. It was designed to gain a broad overview of perceptions on multiple facets of the conference rather than explore each area in depth, so as to inform the design of future, in-depth studies. To facilitate this, the guide included multiple questions covering a range of areas: background information, general information on PASH, strengths/enablers, weaknesses/barriers of PASH, questions pertaining to the key presentations, topics and Community Forum, psychological and sociological perspective of PASH and recommendations. The interview guide was pretested and pilot tested with author SWP who had attended the conference previously as a parent and was then refined prior to use with participants. Interviews were conducted by BCSC with 13 consenting key stakeholders of the PASH Conference. A variety of all key stakeholders were selected. The interviews were conducted either face-to-face or by phone. Interviews were recorded and transcribed verbatim. Interview data was de-identified prior to analysis. It was later noted that parts of the interview guide were leading especially in the strengths section which may have resulted in more positive data.

#### Subjects and recruitment

The co-chair of the PASH Conference recruited potential participants after consultation with the advisory group members. A participant information form was sent to potential participants. Contact details of voluntary consenting participants were provided to the primary researcher who subsequently obtained informed consent and conducted all interviews. No people under the age of 16 were included in the study.

### Data analysis

Thematic analysis, as described by Braun and Clarke, was conducted [24]. Organisation of data analysis was managed in Microsoft Word. The first two interviews were reviewed in detail by two researchers and a draft coding hierarchy was developed individually before being refined through discussion. Author BCSC then read all transcripts and further developed the coding framework. Author SWP and VH were consulted throughout the process. The framework was further refined through collaboration between researchers and tested for fit to the data through a first-cycle coding. The researchers discussed and adjusted the codebook throughout the process. Themes were generated and thematic summaries developed.

### Ethical approval

The study was approved by The University of Wollongong and Illawarra and Shoalhaven Local Health District Health and Medical Human Research Ethics Committee (NO: 2016/714).

## Results

### Participants

Thirteen interviews were conducted either face-to-face or by phone. There did not appear to be any difference in the quality of interview content across interview types. Each interview lasted on average 45 min. Subjects included 2 teachers, 2 parents, 2 youth conference workers, 2 conference presenters, 2 organisers, and 3 PEEPs (Table 1). The PEEPs had received training prior to the conference and became co-facilitators. PEEPS were aged between 16 and 17 years and adult participants ranged between 38 and 60 years.

**Table 1:** Participant demographic details and interview type

Participant ID number	Stakeholder position	Gender	Interview Type
ID01	Conference Organiser	Male	Face-to-face
ID02	Conference Organiser	Female	Face-to-face
ID03	Conference Worker	Female	Face-to-face
ID04	Conference Worker	Female	Face-to-face
ID05	PEEP	Male	Face-to-face
ID06	PEEP	Female	Face-to-face
ID07	PEEP	Male	Face-to-face
ID08	Conference Presenter	Female	Face-to-face
ID09	Conference Presenter	Male	Face-to-face
ID10	Parent who attended conference	Female	Face-to-face
ID11	Parent who attended conference	Female	Phone
ID12	Teacher who attended conference	Female	Phone
ID13	Teacher who attended conference	Female	Phone

*Abbreviations: ID* Identification, *PEEP* Peer Educator engaging Peers

### Thematic analysis

**Fig. 2:**
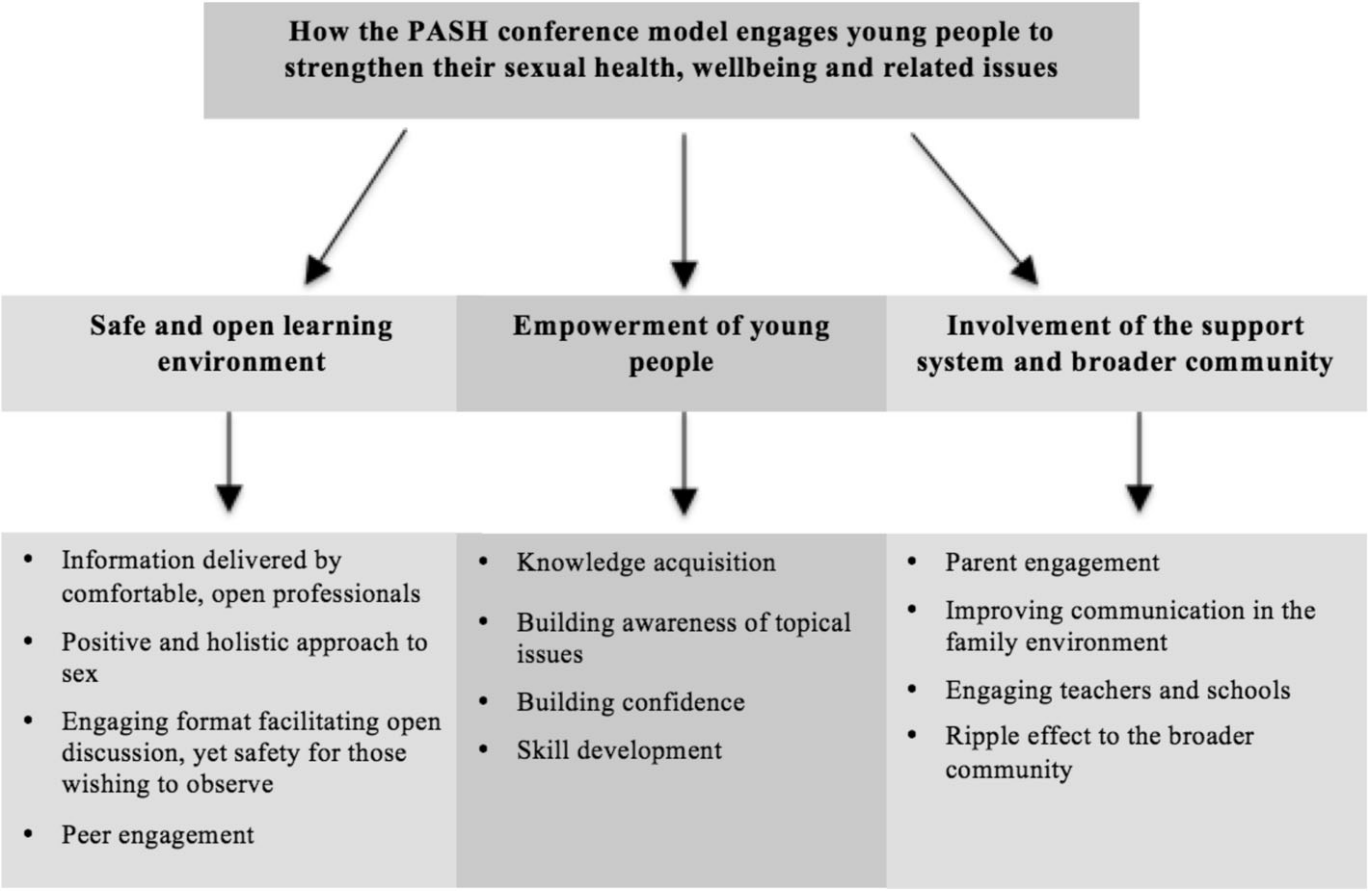
Overview thematic analysis for Part 1

The thematic analysis revealed a range of factors perceived by key stakeholders to have engaged young people attending the PASH Conference to strengthen their sexual health, wellbeing and related issues. These followed three overarching themes (safe and open learning environment, empowerment of young people, and involvement of the support system and broader community) each containing sub-themes as outlined in Fig. 2.

Participants identified a range of recommendations to improve the effectiveness and reach of the conference, following two themes (improving conference format and planning, and enhancing stakeholder engagement) each containing sub-themes as outlined in Part 2: Recommendations.

### Part 1 Engaging young people

#### Safe and open learning environment

All interviewees identified that having an environment conducive to learning at the PASH conference was central to youth engagement. The majority of interviewees expressed that the environment was “comfortable”, “respectful”, “safe”, and “open” enabling young people to relax and discuss sexual health and sex-related issues;

“*It offers a forum for them to be able to talk about all things sex and sexuality and gender without any of the restraints that might be there in other circumstances whether it’s with parents or with schools or even headspace, and other professional forums*” Organiser, ID01.

##### Information delivered by comfortable, open and skilled professionals

Most identified that presenters and facilitators were comfortable, open and at ease with their topic areas. Some interviewees expressed that presenters’ comfort with discussing topics and their ability to engage young people was due to their level of expertise, as well as the light-hearted atmosphere set at the conference. This offered something beyond school-based sex education:

*“You’ve got people there that are more open in their sexuality. Where as you wouldn’t expect that in a school situation. The presenters are more comfortable being themselves and talking about their experiences”* Teacher ID12.

*“School makes it feel a bit uncomfortable, PASH made it more comfortable. I think just having a sense of humour, they were always funny, and they just really made us feel good about it”* PEEP ID06.

A few interviewees identified that having professionals who were comfortable discussing sex and sexual health was a positive way of normalising discussions around sex-related topics. Others expressed the value in having presenters and workers who were light-hearted, friendly and approachable. Some, including PEEPs, perceived it to be crucial that they had “respect” for young people, achieved by being non-judgemental and encouraging attendees to share their experiences, opinions and questions. Some identified that key to creating an engaging environment was having contributors who were passionate about young people and their sexual health.

##### Positive and holistic approach to sex

All interviewees identified that the positive approach taken towards sexual health was a great strength in engaging young people. Organisers and workers tended to describe this as a “normalisation of sex”, where sex is not “pathologised”, and not made a moral dilemma. Most felt this was facilitated through content, delivery, and the presence of PEEPs and the Youth Theatre.

*“Sex is delivered as something very normal and natural, it’s not processed but it’s certainly not fear based at all. So it doesn’t lead with that harm minimisation approach around STIs or pregnancy. Whilst they are aspects of the PASH program, really the way the framework is presented is looking at their overall wellbeing and sexual health being a part of that.”* Organiser, ID02.

Many interviewees, including all PEEPs described the approach to sex as open and non-secretive, consequently facilitating discussion, and the opportunity to learn and form opinions based on a diverse range of material;

*“This is a conference where everything is out there for you now, you can say what you want, you can tell them... Nothing is hidden in the dark. Everything is expressed and almost out there for them to decide”* PEEP, ID05.

##### Engaging format facilitating open discussion, yet safety to those wishing to observe

The conference format included large group sessions and 13 small group ‘hot-topic’ sessions of 15 min duration, which students rotated through. Many expressed that this interactive and speedy small group format was engaging and appropriate for young people.

Some interviewees identified that the format provided an opportunity for discussion, which was still safe for those not wishing to talk in a large group and preferring to engage through observation.

*“It gives them a chance to ask questions in a safe environment. We can always teach them at school, but being teachers they might not want to open up to us and they’re more likely to do that in a group and even if they don’t ask those questions, they may hear questions that other kids have asked.”* Teacher, ID12.

##### Peer engagement

Peer engagement was one of the strongest factors in creating a comfortable and relatable environment. PEEPs were involved in facilitating engagement of young attendees. Almost all stakeholders discussed the positive impact that PEEPS had on learning, by increasing engagement through the creation of a more relaxed, comfortable and familiar environment and a more relatable peer-based role model.

*“It is difficult to engage young people and so that pro-social modelling that can happen from their peers is paramount. Otherwise you have a bunch of adults standing around telling a bunch of young people what to do and they hear that all the time. When they see their peers which they may identify with, they provide a kind of role model for their peers to get involved”* Organiser, ID01.

Peer educators themselves noted that young people are more likely to talk about sex with people they can relate to;

*“They see all these doctors and professionals and they think they’re some big thing and they cannot relate in some way. However with us, we’re their age, they can communicate better and it’s not lost in words”* PEEP, ID05.

A local Youth Theatre (YT) also held performances, covering a range of sexual health issues such as consent, negotiation, protective behaviour and sex and disability. A few interviewees found this to be the greatest strength of the conference. Many expressed that it provided a “relatable”, “real”, “expressive” and “powerful” form of information delivery. Parents noted that it enabled exploration of topics not discussed at home.

*“….it’s a powerful performance illustrating emotion… stuff that I wouldn’t talk about with my son”.* Parent, ID11.

#### Empowerment of young people

##### Increasing sexual health literacy

All stakeholders discussed that acquiring knowledge on a broad range of topics strengthened young people’s sexual health and wellbeing. This extended beyond sex to include knowledge around relationships, mental health and wellbeing. Sexual health literacy was developed through the diversity of content and different tools including the youth forum, hot-topics, the theatre piece, peer education and practical activities such as ‘Embodied Consent’ and ‘Mind, Body and Soul’, which included art therapy and nutritional education.

Many, including teachers, perceived that having speakers who specialised across a variety of areas gave young people a deeper understanding of sexual health than that offered in the Australian School Curriculum. PEEPs found it valuable to have different information sources;

*“Definitely having all those speakers. So you saw the legal side, the sexual therapy side, you saw where everyone was coming from not just like with a teacher, they just say…this is how everything is. Where as when you get a lot of speakers specialising over lots of different areas you can almost put a picture together and understand a lot in depth.”* PEEP, ID05.

Many felt that the PASH Conference addressed important gaps in sex education. PEEPs perceived the conference provided a source of information on check-ups, STIs, safe sex, positive relationships, and other issues that weren’t addressed adequately at school or at home.

*“This is learning more about sexuality, and better sex, at school they don’t talk about things like making sex good or relationships, a little bit about STIs but not much. Check ups and things like that we never learnt much about” PEEP, ID06*

Some stakeholders noted that many families rely on schools to provide sex education to their adolescent. Parents identified the value in PASH addressing a range of topics that they understood would be difficult to cover during school sex education or in the home.

*“It’s the variety, all the topics, they cover a whole lot of issues that you probably wouldn’t be able to cover in one sexual health education. It’s like.. I couldn’t cover all of these topics with my child”* Parent ID10

Teachers also noted the conference highlighting topics that would be of value to introduce in school-based sex education programs;

*“They <the students> were discussing it on the way back and were saying,…we’d like to see more of this, and this is good because of this, so they spoke to me about what they liked*, *and what they thought other people would like to hear”* Teacher ID12

##### Building awareness on pornography, sexting and other topical issues

Many identified that the conference built awareness of topical issues, e.g., consent, legalities around sexting, pornography, mental health, issues faced by those identifying as LGBTIQ, gender, sex and disability, sexuality, relationships, and negotiating better sex. Parents, PEEPs and presenters emphasised that exploring legalities around consent and sexting were particularly important, having broad impacts on younger members of the community. One presenter discussed concern about young men over 16 years being unaware that it is a sexual offence to be involved with a young person under the age of 16.

*“At law, children under the age of 16 can’t consent to any sort of sexual intercourse or touching and that’s quite out of step with what’s happening in the community and we’re finding generally young men are effectively being criminalised when something goes wrong in the relationship”* Presenter ID08

It was felt that the ‘What’s Legal, What’s not’ hot-topic session offered young people a space to discuss differences between legal obligation and actual practice in the community and build awareness of legalities surrounding consent.

Many discussed the value of discussing issues around social media and sexting, making young people aware that they are leaving evidence trails, and potentially procuring child pornography if they have received a nude picture from a person under the age of 16;

*“I think the social media one was almost an eye opener for some people. It definitely informed of the ‘Do’s and Dont’s’. It was about sexting and how it could affect someone if it ended up as child pornography”* PEEP, ID05

Building awareness around pornography and media images appeared to be important. This was covered within a piece from the youth theatre which was felt to provide a light-hearted and relatable means to engage young people, as well as a hot topic session which included interactive exercises to brainstorm differences between ‘porn sex’ and ‘real sex’. The brainstorming session was perceived to explore the negative impact that pornography can have on sexual behaviour due to 1) unrealistic images, 2) lack of consent shown and 3) lack of safe sexual behavioural practices.

The conference was perceived to build awareness of the potential harms of messages received about sex in the media, and the influence and impact that this has on young people, their self-esteem, and sex choices. A couple of interviewees outlined that increasing awareness on pornography would likely have a valuable impact for young attendees by developing their capacity to recognise both the harms and benefits of pornography use. This in turn would likely build their capacity to make informed choices regarding both pornography use and real-life sexual experiences. Gaining awareness around pornography was perceived to be an important decision-making tool at an age when young attendees may not otherwise have the capacity to distinguish between reality and fiction.

##### Building confidence

Most perceived that the conference helped young people develop confidence, enabled by their increase in sexual health literacy, the positive approach towards sex, and the safe learning environment. Some cited that providing young people with a “healthy dose of reality” by highlighting the unrealistic nature of media images and pornography improved self-confidence. Some expressed that confidence was built through the practical activities; while others perceived benefits in young peoples’ opportunity to select individualised information via the service stalls without their parents. Meeting approachable community and youth service stall holders such as Headspace, The Family Centre, Connecting Home program, FSG, and Family Planning NSW, was identified as an opportunity to break down barriers to accessing services such as STI testing, treatment and mental health, services which may have previously been seen as difficult or intimidating to access.

Many felt that PEEPs and YT members built confidence due to the training they received, and their peer educator roles. PEEP interviewees felt they had experienced a transformation in self-confidence.

*“When I started I was always the shy one who sat at the back and didn’t want to talk. But then when I started going along, I became more confident and I started talking to more people and became more open about it. And I’ve had people come up and ask me stuff and I know how to answer it now. It’s good, it has helped.”* PEEP, ID07

##### Skill development

Skills perceived to have been developed through the conference are outlined in Table 2. Skill development was enabled through workshops, discussion, hot topics, information, pamphlets, links to online resources and practical activities (e.g. putting a condom on a banana).

**Table 2:** Skills perceived to have been developed at the PASH Conference

Making informed choices based on what feels right to the individual	
Protective behaviours	
Negotiating better sex	
Skills around consent	
Ability to discuss emotions and sex- related matters with peers and parents	
Accessing services	
Decision-making	
Communication	
Knowledge transfer	
Help seeking	
Critical thinking	

Skills around consent and negotiation were developed through the hot topic run by a local lawyer, and the hands-on concurrent session ‘Embodied Consent’ which was a learning experience of setting boundaries around consent and negotiation whilst having their hand massaged by a peer.

*“The Embodied consent was run by a sex therapist and that is giving them an active experience of setting boundaries and giving themselves permission to state what is right for them…So they do that in the context of the hand massage, so you go into details of saying exactly how you like it, so it challenges people’s notions, their right to speak up, valuing themselves, their assertiveness…”* Organiser, ID02

PEEPs were recognised to have developed particular skills, as outlined in Table 3. This was perceived to be highly empowering and of value to their school community.

“*They have their own couple of days of training, so the more informed and comfortable they are, the more they can support other young people. So they on a general level go back into their school community and peer community with that training”* Organiser, ID02

**Table 3:** Additional skills acquired by PEEPs

Knowledge acquisition and research	
Assistance and co-facilitation of information delivery and discussion	
Peer support	
Knowledge transmission	
Team work	
Leadership	

#### Involvement of the young person’s support system and broader community

##### Engaging parents and families

Parents who had attended valued exposure to the experiences of young adults and the information made available to adolescents. Parents were particularly interested in understanding legalities around sex, online sex, the media, sexting, bullying, and safe sex.

*“I got to see the interesting portrait of what teenagers experience, you know, what goes on, their inner dialogue so it was enlightening being confronted with what they deal with particularly as things have changed over the decades.”* Parent, ID11

The Community Forum was seen as an important adjunct to the conference to engage parents, teachers and the community. However, whilst the forum was perceived to be an important opportunity to engage parents, many perceived engagement inadequate due to poor attendance. Some, including a PEEP, discussed the importance of having youth attend the conference without their parents to facilitate open and free discussion amongst youth unhindered by parent presence.

Several participants, including PEEPs and parents, expressed that the conference increased communication between young adults and their families on sexual health related topics. PEEPs and parents expressed that the conference had made these conversations less uncomfortable.

*“I mean just for us as a family it’s really great that there is this forum that we can talk about anything basically now and feel comfortable and not awkward”* Parent, ID11

##### Engaging teachers and schools

Engagement of teachers was identified as contributing to youth engagement. Teachers brought students to the conference and then attended the hot topic sessions in a ‘teacher only group’. Teacher engagement was perceived to be important given their role in discussing sex and related topics at school. One presenter mentioned that whilst attendance was good, there was a possibility that the conference was attracting teachers who were already more open and informed rather than those in need of exposure to PASH information. However, one teacher said that she had discovered new material that she was considering for her school sex education program. She also perceived that there were teachers present to whom PASH material was new.

*“…there were a few things that I discovered and I thought that would be good to introduce into our program. It was a bit alarming to hear some of the others <teachers> involved weren’t aware of these things so I think it’s really good for those people to be at the conference.”* Teacher, ID12

##### Community impact

Extending the impact of the conference into the broader community was perceived to be important in engaging young people more broadly. Approximately half identified that by creating a space where young people start engaging in dialogue, the conference was initiating a process which would create a ‘ripple effect’, leading to greater awareness of sex and a broad range of related topics that needed to be addressed including sexuality, gender, mental health and wellbeing in the school and broader community. Some also identified the importance of this process being ‘youth-driven’.

*“I think what it’s doing is kind of saying to the community…hey this is an issue that we need to address and it’s also providing a vehicle for young people to be actively involved in that process. Kids go back to their schools and go…hey, guess what we did yesterday… and there is a bit of a ripple effect back into their schools and their communities.”* Presenter, ID09

A few interviewees also expressed that the flow-on effect to the community was positive in that it challenges distorted societal views and may generate positive cultural norms for young people.

*“At a community level it creates a space where young people start having a dialogue, a discussion about consent and what it means to them, They also start to have a discussion about other topics, pornography, sex and disability”* Organiser ID01

### Part 2 Recommendations

**Fig. 3:**
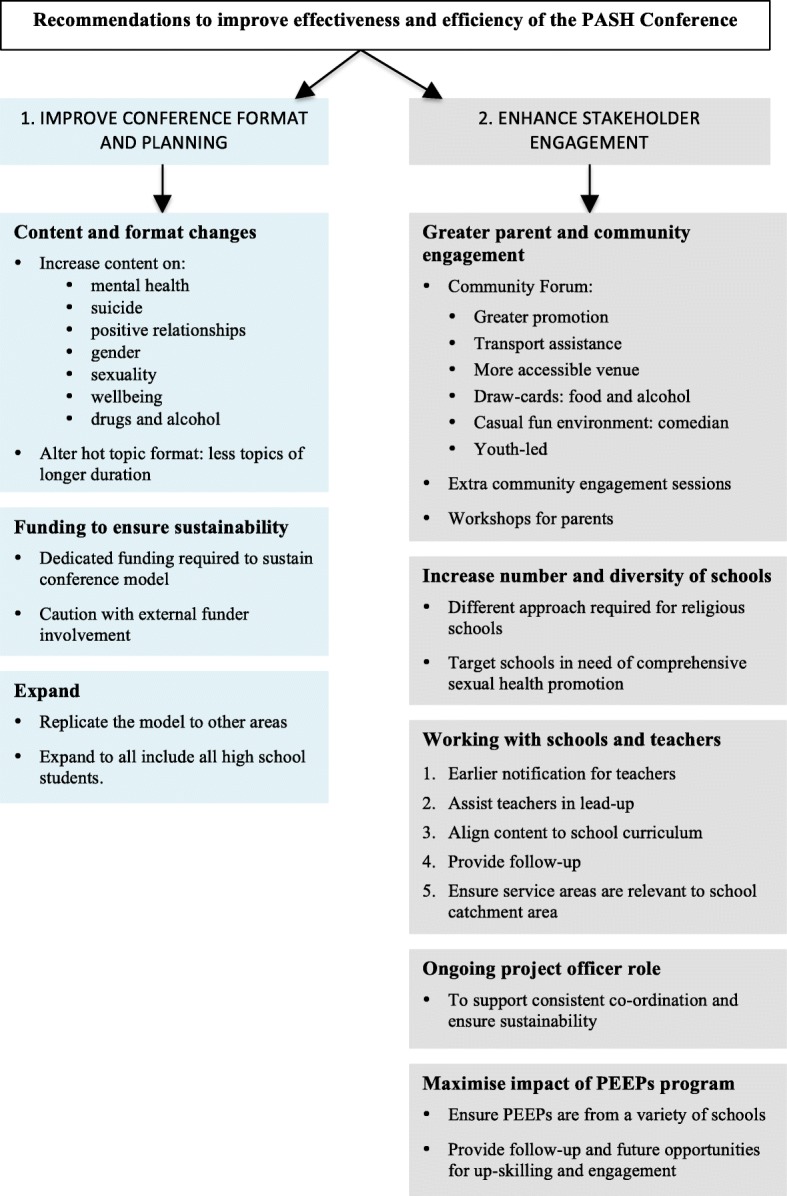
Overview thematic analysis for Part 2 Recommendations

A number of recommendations were identified to improve effectiveness of the conference. These followed two themes 1) improvements to format and planning and 2) enhancing stakeholder engagement (Fig. 3).

### Improve conference format and planning

#### Content and format changes

Most stakeholders identified topic areas that they perceived required increased coverage at the conference. A few interviewees recommended increasing mental health content, highlighting the need to cover mental health within the context of sexual health. One teacher noted the importance of such discussions at this age;

*“There could always be more focus on mental health and mindfulness, more more more more more, because I think what’s happening is we have a lot of kids with anxiety that don’t know how to manage it and they get through school, they get to year 10 and that’s when drugs, that’s what settles them...”* Teacher, ID13

A few recommended increasing content on suicide and bullying, noting that whilst there had been a wave of suicides in the area, young people were receiving minimal dialogue at school and in the home.

*“It’s a very hard conversation to have”….“it’s a real issue in this community. It is a growing issue that we really need to address”* Presenter, ID08

Teachers and workers recommended increasing content on positive relationships, as well as exploring certain topics in greater depth e.g., negotiation, conflict resolution and avoiding abusive relationships.

*“young people’s feedback included how they would like to see more around positive relationships… they need to see what good relationships and positive relationships look like”* Worker, ID04

Increasing content on gender, sexuality, wellbeing, communication, drugs and alcohol was also recommended.

Most expressed that whilst the hot topic format itself was engaging, the 15-min duration of each topic was too short. Many recommended having less hot topics so that each could be of longer duration. Other interviewees however saw benefit to this ‘speed-dating’ approach.

*“ I think there could always be more time, but I think the way that it is set up is to keep it moving quickly, because that’s engaging.*” Worker, ID03

#### Funding to ensure sustainability

Organisers, workers and presenters expressed the need for dedicated funding to sustain this conference model. The current model is based on contributors all working voluntarily bar one short term paid position due to a lack of funding. It was identified that an increase in funding could translate into increased parent and community engagement and incentives could be provided to expert presenters, workers and organisers. Conversely, some interviewees noted potential issues with external funding. This included losing control of the conference to those externally involved and as a result losing what makes PASH unique as a sexual health promotion model. Some discussed that external funding could lead to unnecessary bureaucracy that would take that spontaneity away.

*“ It <PASH> clearly needs funding. That whole funding is a difficult issue as well….sometimes when things are funded they can get caught up in the bureaucracy that then takes that spontaneity away. So I think that needs to be carefully thought about so it maintains a connection with the group that it’s intending to service and that’s really important”* Presenter, ID08

#### Model replication and expansion

Some suggested that this model be expanded and replicated in other regions due to the value it held in strengthening adolescent sexual health. Some interviewees including a teacher and both youth workers, considered that expanding the model could improve adolescent sexual health.

*“There could be a PASH conference for year 7s, year 8s, year 9s, year 10s so they’re getting something like that where all of this information is available you know and it could be modified, but having something where it’s not dumped on the shoulders of the PD/H/PE staff members”* Youth Worker, ID04

*“Looking at the syllabus and making it <PASH> an integral part, somehow making it so schools can embed it into their syllabus’ in some way.”* Teacher, ID13

### Enhance stakeholder engagement

#### Parent and community engagement

Many identified that parents and other community members were not adequately engaged through the conference. The Community Forum event, which is held on the middle night of the conference, aims to engage parents and other community members to discuss sexual health related matters amongst young people. Most perceived that this was ineffective due to poor attendance rates at the forum. Suggestions identified for improving the forum to increase engagement are outlined in Table 4.

**Table 4:** Suggestions to improve the Community Forum

1. Greater promotion	
2. Transport assistance for parents located at a distance from the conference site	
3. More accessible venue - one interviewee mentioned holding the community event at the pub	
4. Using ‘draw cards’ – such as food and alcohol	
5. Create a more casual fun environment, e.g. incorporate a comedian	
6. Have young conference attendees assist in running the forum	

Some interviewees also discussed the value in adding an interactive workshop for parents during the conference. Online workshops were also suggested, including topics such as young people and sex, legal ramifications, accessing services, STI checks, and addressing attitudes and beliefs of adults around sex.

*“I think like a chat group or an online workshop could be really good...yeah, I feel like there needs to be more discussion and I feel like I’m a little bit limited to the friends that are around me and so when some funny antic comes around we talk about it but actually to cover a lot through wider, broader community views or experiences wouldn’t be a bad idea” –* Parent ID11

#### School engagement

Some discussed the importance of expanding the program to involve religious schools, as well as those schools deemed to have an increased need for a comprehensive sexual health program.

*“It would be really exciting to have other high schools get on board and that’s not necessarily <a> sustainability issue, but you know I mean we want to educate as many people as possible if the resources are there”* Youth worker, ID3

*“I would love to see more Catholic and religious schools involved. That’s still a bit of an interesting area.”* – Youth worker, ID4

Teachers identified certain issues, which if addressed, could enhance engagement of young attendees as well as teachers. These are outlined in Table 5.

**Table 5:** Recommendations to enhance teacher and school engagement

1. Earlier notification for teachers	
2. Assist teachers in the lead-up to the conference	
3. Align content to school curriculum	
4. Provide follow-up to build on knowledge, skills and confidence gained during the conference, including a PASH follow up visit in the class room, a phone app or continuing professional development for teachers	
5. Ensure service providers are available for each school catchment area	

#### Engagement of PEEPs

Some noted there would be value in ensuring that PEEPs are trained from all schools that attend. This would mean that each school community benefits from newly acquired skills in peer education.

Almost half of the interviewees expressed the need for increased follow-up and up-skilling of PEEPs post-conference. Mentoring programs and workshops led by PEEPs were provided as examples for continued engagement for PEEPs.

*“I would like to see a little mentoring group maybe come of it, so those students could then come back into the schools and then maybe, like a peer support kind of thing…students to run little programs or have little workshops or you know, be observant and proactive in the classroom and the school” –* Teacher ID13

#### Ongoing project officer role

Organisers expressed that having one paid position at the most recent conference was highly valuable, as it supported consistent co-ordination and delegation of the conference and ensured sustainability.

*“ I think something that worked really well was they created a paid position …and that certainly helped, you know, having someone with some dedicated hours. Because it is it’s a lot of extra work”.* Youth Worker, ID04

## Discussion

In this study, we carried out an introductory investigation into stakeholder perceptions of how the PASH Conference engages young people to strengthen their sexual health and wellbeing, and aimed to identify recommendations to develop the conference. This was intended to provide early research to inform the design of future in-depth analyses including evaluation of behavioural outcomes, as well as provide introductory information for public health educators on a new conference format for adolescent sexual health education. Key stakeholders identified that the PASH Conference engages young people to strengthen their sexual health and wellbeing through three predominant ways: having a safe and open learning environment, empowering young people, and involving the support system and broader community of the young person. These themes have emerged frequently in literature outside of Australia exploring positive youth development models that have been effective in promoting adolescent sexual health outcomes [19, 20].

Having a safe and open learning environment appeared to be key to engaging young attendees at the conference. Creating a supportive, positive atmosphere has frequently been documented in the literature as instrumental to effective adolescent sexual health programs [20]. Receiving information from sexual health professionals who were open and at ease, and who had ‘respect’ for young people appeared to contribute significantly to establishing this atmosphere. Stakeholders perceived that having presenters who were comfortable in their own topic areas normalised discussions around sex-related topics, and provided a more effective means of sex education than school based sex-education. Understanding the intricacies of what made these presenters more open and comfortable as teachers in sexual health and wellbeing was not explored during interviews. It may be because the presenters are subject experts and having confidence in their own knowledge base allows them to be more open and comfortable in their respective topic areas. It would be beneficial to further explore this in future research and to evaluate whether these qualities or skills could be taught or transferred to school-based sex educators. Gaining further insight into qualities of effective teachers of sex-education is highly relevant, given that 50% of young Australians express significant dissatisfaction with sex education at school, with many feeling that teachers are not generally suited to carrying out this role [2].

Peer engagement was also deemed key to creating a safe learning environment, enabled through PEEPs, the Youth Theatre and a peer social environment. Having peer-based role models made discussions more relatable, and it was identified that young people were more likely to engage, and increase their knowledge, skills and awareness surrounding sexual health. Peer-led sexual health interventions frequently demonstrate that peer educators and the young people they are teaching perceive benefits in terms of skill and knowledge acquisition [25]. However, whilst peer-led programs frequently report increased youth engagement, there is mixed evidence for their actual impact on sexual health outcomes [14–18]. For example, a recent literature review of adolescent peer-led HIV education programs demonstrated effectiveness in improving knowledge, attitudes, beliefs and self-efficacy however the studies were equivocal on changes in sexual behaviour [15]. Another recent systematic review also demonstrated that programs with a peer-led approach to youth sexual health education appear effective in changing knowledge and attitude, but not behaviours [18]. Whilst this study has illuminated that peer education was perceived to be a key factor at the PASH Conference in creating an environment that was conducive to increasing knowledge and skills, it was an introductory qualitative analysis. Given the unclear impact of peer-led programs on sexual health outcomes in the literature, and as this current research is an introductory evaluation of the PASH conference, it is recommended that this relationship be further explored in future evaluations of the conference. Broadening the study sample to young conference attendees and evaluating behavioural outcomes would be of particular benefit.

Empowering youth was also seen as key to engaging young people to strengthen their sexual health and wellbeing at the PASH Conference. Youth empowerment has frequently been cited in literature on effective PYD programs [19, 20]. Stakeholders perceived that this was enabled through improving sexual health literacy, building awareness, confidence and skills. Knowledge and awareness was perceived to have been acquired in a broad range of areas including wellbeing, mental health, consent, legalities around sexting, pornography, positive and negative relationships, STIs, contraception and more. This is important as Australian youth report that these topics are desired yet missing in school-based sex education [2, 6]. Australian students have identified that sex education often feels irrelevant to real-life experience, with inadequate discussion of important issues [2]. Stakeholders also identified areas requiring increased coverage at the conference, such as mental health, suicide, bullying, gender, sexuality, drugs and alcohol, and positive relationships. We suggest that increasing content on these areas may be of value for future adolescent sexual health conferences and other sexual health promotion programs, and that this should be further explored in future studies.

Building awareness and developing skills in evaluating the potential harms of pornography and sexual media content through an interactive hot topic session and through the youth theatre was perceived to be highly valuable. This is understandable given that new technologies have expanded adolescents’ access to pornography and online sexual media content. International research demonstrates that online pornography exposure is common among young people [26]. Research links greater exposure to sex in the media with changes in sexual outcomes and attitudes [27]. Recent literature has demonstrated an association between consumption of greater amounts of sexual content and earlier age of first sexual intercourse [28, 29] earlier pregnancy [30], and greater acceptance of casual sex [31]. Online media exposure to sexual content is much more difficult for parents to monitor than through traditional mediums [27]. Our results indicated that stakeholders identify that the PASH conference provides a forum to develop awareness and skills in critiquing pornography and sexual media content. It was perceived that this would increase young people’s ability to recognise the harms and risks of regular pornography use and would increase their capacity to make informed choices in this domain. Given outcomes of high levels of exposure to sexual media content, in combination with perceived inadequacies of school sex education in Australia, the PASH conference appears to offer a potentially effective means of addressing these areas of concern. Further evaluation of the impact of the conference, including assessment of behavioural outcomes would be of value.

Increasing awareness of legalities around sexting and consent was also deemed particularly valuable by parents, peer educators, teachers and presenters. Sexting involves the exchange of sexual material (images or text) via mobile phone or internet [27]. In a nationally representative survey in Australia over half of adolescents had received sexually explicit text messages [2]. From a developmental perspective, sexting can be a normal form of intimate expression of sexuality through new technologies [32]. However there are also various risks associated with sexting including image-based abuse, technology-facilitated sexual violence, and content being sent under coercion [33]. Other risks include material being passed on to a third party as a method of bullying, and there is potential for youth senders to be prosecuted under child pornography laws [34]. Peer educators and teachers in the present study noted that discussion around the potential consequences of sexting as well as legalities surrounding consent was eye opening and particularly relevant in our current context where sexting is common yet the impacts are often unclear to both senders and receivers. Recent literature has suggested a need for risk-reduction interventions surrounding sexting [27]. This introductory study suggests that the PASH conference may provide an effective risk-reduction intervention for these increasing areas of concern, i.e. sexting, consent, online pornography use and exposure to sexual images in the media. Once again, further research is required to evaluate the conference’s effectiveness in greater depth in this domain and to assess behavioural outcomes.

Building confidence and developing skills were deemed to be strong factors contributing to youth empowerment, characteristics that are often reported in the literature as important qualities of effective PYD programs [19, 20]. Our results demonstrated that stakeholders perceived that major contributors to this were the positive approach to sex, the hands-on activities and increase in sexual health literacy. Many discussed that this was particularly evident for PEEPs and that these adolescents were able to return to their school and peer communities with the skills and confidence they had developed in listening to, supporting and educating others in topics they may have previously felt uncomfortable discussing. Peer educators themselves noticed a considerable boost in their self-confidence and expressed that the skills they had gained in peer support had been of lasting benefit. Previous literature has identified that peer educators report increases in self-efficacy, self-esteem and ability to communicate with others about sexual issues [35]. Peer educators report increased knowledge, awareness of their own health, and concern about the health of others [36].

Our introductory findings suggest that developing the skills and confidence of individuals through adolescent sexual health programs such as the PASH Conference may have positive implications not only for the individual, but their respective school and peer communities given their ongoing peer support and peer education roles in these contexts. To maximise this impact on the individual and their school communities, some stakeholders recommended that PEEPs be drawn from every school attending the conference, and that follow-up and future up-skilling of PEEPs be enhanced. However, as previously discussed, whilst peer-led interventions have demonstrated effectiveness in increasing knowledge and changing attitudes, they have been equivocal in terms of actual outcomes [15, 18]. For this reason, it would be of value to evaluate behavioural outcomes using a broader sample to further investigate the perceived positive implications of this intervention, as well as provide further justification to follow these recommendations.

Participants perceived that support system and community involvement was central to engaging young people. This is in line with literature demonstrating that PYD programs that target the family, school and/or community surrounding youth lead to enhanced adolescent sexual health outcomes compared to programs that target youth only [37–39]. It was felt that the conference had improved communication in the family environment, adequately engaged teachers, and led to a ripple effect to the broader community with young people taking information back into their schools and broader communities.

However, many suggested that both community and support system engagement needed improvement. This is important to consider in future conferences and in the design of other adolescent sexual health programs, and should be re-evaluated in future research as models that engage the community and support system well frequently demonstrate effectiveness [9, 10, 12]. Specific recommendations included increasing the number of community engagement sessions, and improving the community forum to enhance engagement of parents and the broader community, e.g. having young conference attendees help run the forum. Providing workshops for parents was also suggested, a tool often found to be of value in effective adolescent sexual health programs [20]. This could provide a means for equipping parents with tools, for example in recognising and managing negative media influences on their adolescents and in facilitating discussion of its impacts – tools recognised in the literature to be highly important [27].

These recommendations are important considerations for future PASH conferences and require further exploration. They echo health promotion concepts of creating supportive environments and strengthening community action through community empowerment, which requires access to information and learning opportunities [22]. Many organisers, workers and presenters expressed the need for increased funding to sustain the conference model. However, some interviewees noted that external involvement through funding could lead to losing control of the conference and losing what makes it unique as a PYD and health promotion model.

Replication of the PASH Conference model into other contexts was recommended by some due to the value they perceived it has in strengthening adolescent sexual health. Expanding to include all high school years was suggested, an important recommendation as programs with evidence of improving adolescent sexual health outcomes are frequently those that deliver content across age groups [39, 40].

### Limitations

Due to the involvement of the co-chair of PASH in participant recruitment, there is potential bias introduced which may have skewed the sample towards those interested in the continuation of the PASH Conference. The standardised interview was only piloted with one stakeholder, a parent. As the stakeholders were of a wide range of ages, professional backgrounds and varying levels of involvement in the conference it would have been of value to pilot the interview with a representative of each stakeholder type.

As this study was intended as early work, the interview schedule was designed to provide a broad overview of how the conference engages young people. As such, it had limited scope to provide in-depth exploration of particular aspects of conference engagement. For this reason, it is intended as an introductory piece of research to inform future, in-depth analyses on how the conference engages young people, recommendations for improvement, and behavioural outcomes. It is suggested that findings and recommendations for improvement be further explored and clarified.

The interview schedule in parts were more leading when exploring strengths of the conference compared to weaknesses which may have biased the findings to give a more positive perspective from the key informants.

Young people attending the conference, who were not peer educators, were excluded from the sample as this was a stakeholder evaluation rather than a consumer evaluation. It is recommended that further evaluation be undertaken incorporating the perspectives of young conference attendees, as well as measuring behaviour change outcomes.

## Conclusion

An innovative PASH Conference program modelled on health promotion and PYD frameworks and incorporating previously demonstrated effective elements including peer education and involvement of support systems appears promising. This provides an early piece of research to inform the design of future research including evaluation of behavioural outcomes. It also provides introductory information for public health educators to develop a new conference-style program based around positive youth development principles. Description of key elements of the PASH Conference found to be effective in engaging young people to strengthen their sexual health and wellbeing and recommendations identified by key stakeholders to improve effectiveness of the program will be useful to inform future, more in-depth analyses and in considering future development and expansion of the program.

## Additional file

Additional file 1: Interview Guide (PDF 94 kb)

## Abbreviations

ID: Identification
PASH: Positive Adolescent Sexual Health
PEEPs: Peer Educators engaging Peers
PYD: Positive youth development
STIs: Sexually transmitted infections
WHO: World Health Organization
YT: Youth Theatre
