# Supplementary material for: A positive approach to adolescent sexual health promotion: a qualitative evaluation of key stakeholder perceptions of the Australian Positive Adolescent Sexual Health (PASH) Conference
Source: BMC Public Health. 2019 Jun 3;19:681. doi: 10.1186/s12889-019-6993-9 (PMC6547521; doi:10.1186/s12889-019-6993-9)
Supplement: Supplementary file 1 — Interview Guide (PDF 94 kb) [file 12889_2019_6993_MOESM1_ESM.pdf]

Interview Schedule

Background information on interviewee	• Male/ female/other<br>• Age: ______ (years)<br>• Involvement in PASH conference: Organiser/ presenter/ teacher/ PEEP/ worker
Strengths/ enablers of PASH	• What in your opinion were strengths of the conference? *(Prompt when required: Can you give an example?)*<br>• How did the conference enable engagement of **young people**?<br>• How did the conference help young people increase their **knowledge** on:<br>• Sexuality and sexual health<br>• Related services (mental health/drug and alcohol)<br>• Access to sexual health services?<br>• How did the conference help young people build **confidence** in:<br>• Sexuality and sexual health<br>• Accessing sexual health services<br>• Communicating what they want or don't want in relation to sex?<br>• How did the conference help young people build **skills** around:<br>• Protective/Safe behaviour<br>• Reduction of STI risk<br>• Access to services (eg treatment or testing)<br>• Communicating what they want or don't want in relation to sex?<br>• Were the topics covered **appropriate** and innovative in their transmission?<br>• Prompts: sex and disability, legal aspects, where to get help, social media, drugs and alcohol *(Provide the attached list of conference topics to interviewee)*<br>• Was the content **informative** and valuable to young people and their support system?<br>• Was **multimedia and arts** based platforms used effectively for example, engagement of the byron youth theatre?<br>• How do the **PEEPs** (Peer Educators engaging Peers) assist in creating a positive adolescent sexual health program?
Weaknesses/ Barriers of PASH	• What in your opinion were weaknesses of the conference and how can they be improved upon? In terms of: (*take prompts from previous box)*<br>o **Engaging young people**?<br>o **Knowledge** – *use above prompts*<br>o Building **confidence** in – *use above prompts*<br>o Building **skills** around – *use above prompts*<br>• Were any of the topics covered **inappropriate** or **ineffective** in their transmission?<br>o Prompts: sex and disability, legal aspects, where to get help, social media, drugs and alcohol. *(Provide attached list of conference topics)*<br>• Are there elements of the **PEEPs** program that do not work or could be improved?
Questions pertaining to key presentations, topics and community forum	• How do you think the presentations, topics and the community forum **enable** young people and their support system (parents, teachers, workers) to build their knowledge, confidence and skills in the listed topics in dealing with sexual health and related matters? *–provide list*<br>• We had 13 hot topics and 5 concurrent sessions – what do you think was covered effectively? What was done well? What could be improved? *(Ask if not already adequately covered)*<br>*→ Prompt each with printed list of the conference program:13 hot topics & 5 concurrent sessions & community forum*
Psychological and Sociological perspective of PASH	• What do you consider to be the **personal impact** of the conference on young people?<br>o Prompts around interpersonal and intrapersonal impacts:<br>- emotional, intellectual, physical impacts<br>- exploring their own sexuality, gender and sexual health<br>- any suggestions on how this could be improved?<br>• What **impact**/influence do you believe the PASH conference has at a **community level**? Any suggestions on how this could be improved?<br>• Do you think the conference is a **platform** for young people, parents, teachers, services providers and local media to discuss issues, barriers and solutions around adolescent sexual health?<br>- Prompts: Accessability, affordability, appropriateness of services
Recommend - ations	• How can future PASH conferences be **improved**?<br>o To engage young people more?<br>o To improve their sexual health and wellbeing positively?<br>o Topic content?<br>o Program format?<br>• How can we make the conference more **sustainable**? (*Go beyond funding*)

Conference program

13 Hot Topics

- Love and Sex online
- Identity, Gender and Sexuality
- Sex and Disability
- Check Ups and where to get help
- Negiotiating better sex
- Body Mods – tattoos and harm reduction
- Social Media and Sex
- Party Safe (drugs, alcohol, inhibition and risky behaviours)
- If it's not on…It's not on (Condoms as protection)
- Healthy Mind
- Contraception
- Unplanned Pregnancy
- Safter Sex – What's Legal, What's not

Concurrent sessions:

- Let's talk about Sex – with Dolly Doctor
- Byron Youth Teather
- Embodied consent – active listening and communication
- Mind, Body, Soul
- Peer Education Engagement Program

Community Forum
